# Association between baseline and changes in serum uric acid and incident metabolic syndrome: a nation-wide cohort study and updated meta-analysis

**DOI:** 10.1186/s12986-021-00584-x

**Published:** 2021-06-09

**Authors:** Sen Chen, Nianwei Wu, Chuan Yu, Ying Xu, Chengfu Xu, Yuli Huang, Jian Zhao, Ningxiu Li, Xiong-Fei Pan

**Affiliations:** 1 Department of Health and Social Behavior, West China School of Public Health and West China Fourth Hospital, Sichuan University, Chengdu, China; 2 Kidney Disease Center, The First Affiliated Hospital, College of Medicine, Zhejiang University, Hangzhou, China; 3 Department of Gastroenterology, The First Affiliated Hospital, College of Medicine, Zhejiang University, Hangzhou, China; 4 Department of Cardiology, Shunde Hospital, Southern Medical University, Foshan, China; 5 The George Institute for Global Health, Faculty of Medicine, University of New South Wales, Sydney, Australia; 6 The Ministry of Education - Shanghai Key Laboratory of Children’s Environmental Health, Xinhua Hospital, Shanghai Jiao Tong University School of Medicine, Shanghai, China; 7 Division of Epidemiology, Department of Medicine, Vanderbilt University Medical Center, Vanderbilt Epidemiology Center, Nashville, USA; 8 Department of Epidemiology and Biostatistics, and Ministry of Education Key Lab of Environment and Health, School of Public Health, Tongji Medical College, Huazhong University of Science and Technology, Wuhan, China

**Keywords:** Metabolic syndrome, Serum uric acid, Chinese, Cohort, Meta-analysis

## Abstract

**Background:**

To prospectively examine the associations of baseline serum uric acid (SUA) and SUA changes with incident metabolic syndrome (MetS) and update the evidence through a meta-analysis.

**Methods:**

Our analyses were based on the China Health and Retirement Longitudinal Study from 2011–2012 to 2015–2016. The exposures were baseline SUA and SUA changes, and the outcome was incident MetS assessed in 2015–2016. Logistic regression was used to estimate odds ratios (ORs) and 95% confidence intervals (CIs). A meta-analysis was conducted to synthesize evidence from all cohort studies on the same topic.

**Results:**

Of 3779 participants (47.2% men; mean age: 59.5 years) without MetS, 452 participants developed MetS after a follow-up of 4 years. Compared to the lowest quartiles, the adjusted ORs (95% CIs) for MetS were 1.08 (0.77–1.50), 1.32 (0.95–1.82), and 1.55 (1.12–2.16) for three higher quartiles of baseline SUA, and 1.23 (0.89–1.71), 1.39 (1.00–1.93), and 1.89 (1.38–2.58) for three higher quartiles of SUA changes. Each increment of 1 mg/dL of baseline SUA level was associated with 19% higher odds of MetS (adjusted OR 1.19; 95% CI 1.07–1.33). In the meta-analysis of 24 cohort studies among 140,913 participants, the pooled relative risk (95% CI) was 1.32 (1.25–1.40) for the highest versus lowest SUA category, and 1.15 (1.09–1.21) for each 1 mg/dL increase in the SUA level.

**Conclusions:**

Both baseline SUA and longitudinal SUA changes were positively associated with risk of MetS among middle-aged and elderly Chinese, which was supported by findings from a comprehensive meta-analysis across multiple populations. SUA levels might need to be monitored closely for subsequent risk of MetS in clinical practice.

**Supplementary Information:**

The online version contains supplementary material available at 10.1186/s12986-021-00584-x.

## Background

Metabolic syndrome (MetS) is a pathological state of metabolic disorder that comprises elevated blood pressure (BP), dysglycemia, dyslipidemia, and abdominal obesity [1]. Although the criteria for MetS vary slightly between countries, one out of four is estimated to have MetS globally [2]. With high prevalence, MetS contributes to 30–52% of diabetes, 12–17% of cardiovascular disease, and 6–7% of all-cause mortality [3]. In the context of rapid lifestyle transitions, MetS is emerging as one of the leading public health challenges worldwide.

An excessive level of serum uric acid (SUA) can stimulate oxidative stress and inflammation, reduce the nitric oxide bioavailability, and accelerate the development of insulin resistance, which suggests a possible role of elevated SUA in the impairment of metabolic homeostasis [4, 5]. Since MetS is a frequent comorbidity among patients with hyperuricemia, there has been a hypothesis for positive associations between higher SUA levels and incident MetS. This hypothesis was confirmed in a meta-analysis of cohort studies from several countries six years ago [6]. However, most of the included cohort studies were conducted in non-Chinese populations, or only assessed the relationship of one-time baseline SUA level and incident MetS. The only included Chinese study was based on secondary analyses of health check-up data from a single health examination center [7], and large population-based cohort studies on this topic were still sparse in Chinese settings. In addition, two recent studies in other populations that focused on the longitudinal changes in SUA showed inconsistent findings [8, 9]. Since the national prevalence of MetS could reach up to 33.9% among adults in China [10], we intended to revisit the issue using national population-based longitudinal data that contain repeated measures of SUA.

Capitalizing on a nation-wide cohort study in China, we assessed the associations of baseline SUA and SUA changes with risk of MetS among middle-aged and elderly adults, and examined whether the associations differed across subpopulations. As multiple new cohort studies were published across the globe since the last meta-analysis, we also conducted a comprehensive meta-analysis of all cohort studies to update the evidence on this topic.

## Methods

### Study design and population

The China Health and Retirement Longitudinal Study (CHARLS) is an ongoing national population-based cohort study that is conducted to address aging-related research questions among middle-aged and elderly adults (≥ 45 years) in China. The study design of CHARLS was detailed elsewhere [11]. Using a four-stage, stratified, cluster probability sampling design, 17,708 participants were enrolled from 450 rural villages or urban communities of 28 provinces in China at baseline (2011–2012). Data were collected by questionnaires, physical measurements, and biomarker measurements at baseline and during follow-up until 2015–2016. The cohort study was approved by the Biomedical Ethics Review Committee of Peking University (IRB00001052-11015), and all participants provided informed consent.

Of 17,708 study participants, 10,131 completed questionnaires and provided blood samples at baseline. We excluded the participants due to lack of information on SUA (*n* = 163) or MetS (*n* = 456) at baseline, or presence of MetS at baseline (*n* = 2,551). Of the eligible participants, we excluded participants who did not participate in the follow-up survey in 2015–2016 (*n* = 747), or who lacked information on MetS during follow-up in 2015–2016 (*n* = 2,435). Finally, 3,779 participants were included in our analyses (Additional file 1: Figure S1). The 3182 participants who were excluded were more likely to be older, from urban areas, have lower BMI, and higher SUA levels, but were less likely to be educated, married, and from rural areas compared to the included 3779 participants (*P* ≤ 0.003 for all, Additional file 1: Table S1).

### Data collection

Structured questionnaires were used at baseline to collect information including demographics, socioeconomic status, health-related behaviors, and medical history by trained interviewers [11]. Age was confirmed at baseline (2011–2012). Residence was classified into rural and urban areas. Education level was categorized into illiterate, primary school, and middle school or above. Marital status was categorized into married and unmarried (including separated, divorced, widowed, and never married). Smoking and drinking were grouped as never, former, and current smoking/drinking. Current smoking was defined as having the habit of smoking cigarettes, cigars, tobacco, or pipe currently. Current drinking was defined as having drunk any alcoholic beverages (wine, beer, or liquor) for more than once a month in the past year.

Physical measurements were collected at baseline and the follow-up in 2015–2016. BP was measured three times at 45-s intervals with an electronic sphygmomanometer on the left arm, and an average of three readings was used for analyses. Waist circumference (WC) was horizontally measured at the level of the navel using a soft measure tape. Body weight and height were measured using a digital weight scale and stadiometer. Body mass index (BMI) was calculated as the body weight in kilograms divided by the square of the height in meters (kg/m^2^).

Biomarker measures were completed at baseline and in 2015–2016. SUA was measured by the UA plus method. Fasting blood glucose (FBG), triglyceride (TG), and high-density lipoprotein cholesterol (HDL-C) were measured by the enzymatic colorimetric test. Glycosylated hemoglobin (HbA1c) was measured by high performance liquid chromatography. Prevalent diabetes at baseline was defined as FBG ≥ 126 mg/dL, HbA1c ≥ 6.5%, or self-reported doctor diagnosis [12]. Rate-blanked and compensated Jaffe creatinine method was used to measure serum creatinine. eGFR were calculated using the Chronic Kidney Disease Epidemiology Collaboration Study equation:

$${\text{eGFR}} = 141 \times min {\text{S}}cr/\kappa ,{ }1^{\alpha } \times max\left( {Scr/\kappa ,1} \right)^{ - 1.209} \times 0.993^{{{\text{Age}}}} \times 1.108\left[ {if\;female} \right] \times 1.159\left[ {if\;black} \right],$$

where Scr is serum creatinine, $\kappa$ is 0.7 for females and 0.9 for males, $\alpha$ is − 0.329 for females and − 0.411 for males, min means the minimum of ${\text{Scr}}/\kappa$ or 1, and max means the maximum of ${\text{Scr}}/\kappa$ or 1 [13].

### Definitions of exposure and outcome

The primary exposure was SUA levels at baseline, which was assessed as sex-specific quartiles. In additional analyses, hyperuricemia was defined as a level of SUA ≥ 7.0 mg/dL in men or ≥ 6.0 mg/dL in women [14]. The absolute and percentage changes in SUA over 4 years, assessed as the difference between the SUA levels at baseline and during follow-up in 2015–2016, were the secondary exposure.

The outcome was incident MetS that was diagnosed in the last follow-up among participants who did not have MetS at baseline. MetS was defined according to the criteria from the Chinese guidelines for the management of dyslipidemia in adults [15]. MetS was diagnosed if at least three out of five criteria were met: (1) abdominal obesity (WC ≥ 90 cm in men or ≥ 85 cm in women); (2) elevated FBG (≥ 110 mg/dL) or drug treatment of elevated glucose; (3) elevated BP (≥ 130/85 mmHg) or antihypertensive drug treatment in people with a history of hypertension; (4) elevated TG (≥ 150 mg/dL); and (5) reduced HDL-C (< 40 mg/dL).

### Statistical analyses

Data were summarized as mean and standard deviation (SD) for continuous variables, and as frequency and percentage for categorical variables. Differences of basic characteristics were compared using ANOVA (for continuous variables) and chi-square test (for categorical variables) across sex-specific SUA groups.

Logistic regression was used to estimate odds ratios (ORs) and corresponding 95% confidence intervals (CIs) for associations of baseline SUA with incident MetS. Potential confounders were adjusted for in regression models in a stepwise manner. In Model 1, we adjusted for baseline age (continuous, years), gender (men and women), residence (urban and rural), education level (illiterate, primary school, and middle school or above), marital status (married and single), cigarette smoking (never, former, and current), and alcohol drinking (never, former, and current). Baseline WC (cm), BMI (kg/m^2^), systolic BP (mmHg), FBG (mg/dL), TG (mg/dL), HDL-C (mg/dL), and eGFR (mL/min/1.73m^2^, all as continuous variables) were additionally adjusted for in Model 2. Similar analyses were done when the absolute and percentage changes of SUA during follow-up were assessed as quartiles, and baseline SUA was additionally adjusted for in Model 2. Linear trends across SUA quartile groups were estimated by modeling SUA as the median of each quartile. We conducted subgroup analyses by sex (men and women), age (45–59 and ≥ 60 years), and BMI (< 24 and ≥ 24 kg/m^2^ based on the Chinese cut-offs for overweight and obesity) [16]. A product term of each stratifying variable and SUA groups was added in the main logistic regression model to examine the potential effect modification (interaction) using the Wald test. We conducted a sensitivity analysis to account for confounding from comorbidities by excluding the participants with prevalent hypertension or diabetes at baseline. We also conducted analyses of the association of combined baseline SUA and absolute SUA changes with incident MetS by generating four categories based on their median values (low baseline/minimal increase, high baseline/minimal increase, low baseline/high increase, and high baseline/high increase). In addition, we assessed the associations of baseline SUA and absolute SUA changes with MetS components. SPSS 20.0 (IBM Co., Armonk, NY, USA) was used for all statistical analyses. Two-tailed *P* values less than 0.05 were considered statistically significant.

### Meta-analysis

We conducted a meta-analysis of effect estimates from our study and other cohort studies that examined the relationship between the baseline SUA levels and MetS risk in adults. PubMed, ISI Web of Science, and Embase were searched for cohort studies up to January 30, 2021, using a search strategy that combined MeSH terms and keywords for SUA, hyperuricemia, or gout, metabolic syndrome, and cohort study. Eligibility criteria for meta-analyses included: (1) original cohort studies; (2) assessing the association between baseline SUA and incident MetS; (3) reporting effect estimates (either HR or OR) and 95% CI; (4) conducted in adults; and (5) a minimum follow-up duration of 1 year. The quality of included studies was assessed using the Newcastle–Ottawa quality assessment scale [17].

Basic characteristics and fully adjusted effect estimates were extracted from each individual study. Literature search and data extraction were performed by two investigators (S.C. and N.W.), and any discrepancies were resolved by consensus. Heterogeneity across studies was evaluated using the Q test. Random-effects (when Q test’s *P* < 0.01) or fixed-effects models (when Q test’s *P* > 0.01) were applied to obtain an overall relative risk (RR) for the highest SUA level category compared with the lowest SUA level category and each 1 mg/dL increase in the SUA level. When studies separately reported effect estimates for men and women or different age subgroups, effect estimates were pooled in individual studies by using random effect meta-analysis first, and pooled estimates were combined with those from other studies for the final meta-analysis. If effect estimates were presented as ORs in identified studies, they were converted to RRs using the formula (RR = OR/([1 − pRef] + [pRef × OR]), where pRef is the prevalence of the outcome in the reference group [18]. HRs were considered as approximate RRs for the meta-analysis. Subgroup analyses were done by region (Asian, America and Europe), sex (men and women), mean age (< 45, 45–65, and ≥ 66 years), and median follow-up time (< 4 and ≥ 4 years), and subgroup heterogeneity was assessed by the meta-regression. Publication bias in the meta-analysis was assessed by the funnel plot, Egger’s test, and Begg’s test. Stata 16.0 (Stata Corp LLC) was used for the meta-analysis.

## Results

### Baseline characteristics

Of the included 3,779 participants, the mean (SD) age and BMI were 59.5 (8.7) years and 22.7 (3.3) kg/m^2^. 47.2% were men and 70.1% were rural residents. Overall, the average SUA level was significantly higher in men than in women (5.0 [1.3] vs. 4.0 [1.1] mg/dL, *P* < 0.001). Participants in the fourth baseline SUA quartile group were more likely to be older, urban residents, and alcohol drinkers, and to have higher BMI, WC, BP, TG, and serum creatine compared with those in the lowest quartile group (Table 1).

**Table 1:** Baseline characteristics across quartiles of baseline SUA in the CHARLS

Baseline characteristics	Quartiles of baseline SUA^†^				*P* value*
	Quartile 1 (*N* = 941)	Quartile 2 (*N* = 948)	Quartile 3 (*N* = 944)	Quartile 4 (*N* = 946)	
Men, *n* (%)	445 (47.3)	446 (47.0)	446 (47.2)	446 (47.1)	0.999
Age, mean (SD), years	58.7 (8.5)	59.1 (8.5)	59.6 (8.7)	60.8 (9.0)	< 0.001
BMI, mean (SD), kg/m^2^	22.4 (3.3)	22.5 (3.4)	22.7 (3.0)	23.1 (3.4)	< 0.001
Urban residence, *n* (%)	232 (24.7)	254 (26.8)	320 (33.9)	324 (34.2)	< 0.001
Illiterate, *n* (%)	269 (28.6)	267 (28.2)	245 (26.0)	263 (27.8)	0.665
Married, *n* (%)	837 (88.9)	851 (89.8)	851 (90.1)	829 (87.6)	0.304
Current smokers, *n* (%)	316 (33.6)	298 (31.4)	284 (30.1)	297 (31.4)	0.640
Current alcohol drinkers, *n* (%)	218 (23.2)	224 (23.6)	260 (27.5)	268 (28.3)	0.001
WC, mean (SD), cm	81.6 (8.4)	82.3 (8.7)	82.8 (8.5)	84.0 (9.4)	< 0.001
Systolic BP, mean (SD), mmHg	124.2 (19.1)	125.2 (20.1)	126.5 (20.5)	129.3 (20.5)	< 0.001
Diastolic BP, mean (SD), mmHg	73.2 (11.4)	73.0 (11.6)	73.9 (12.0)	75.0 (11.7)	0.001
FBG, mean (SD), mg/dL^‡^	105.0 (33.6)	102.6 (21.5)	102.7 (19.6)	103.1 (21.0)	0.105
TG, mean (SD), mg/dL^‡^	95.5 (44.1)	97.0 (44.5)	103.9 (58.2)	113.1 (59.5)	< 0.001
HDL–C, mean (SD), mg/dL^‡^	54.3 (14.0)	55.3 (14.1)	55.1 (14.4)	54.5 (14.1)	0.358
Serum creatine, mean (SD), mg/dL	0.69 (0.15)	0.74 (0.15)	0.77 (0.16)	0.85 (0.22)	< 0.001
eGFR, mean (SD), mL/min/1.73 m^2^	99.4 (10.6)	95.1 (11.6)	92.8 (11.7)	85.9 (15.2)	< 0.001

BMI, body mass index; BP, blood pressure; CHARLS, China Health and Retirement Longitudinal Study; eGFR, estimated glomerular filtration rate; FBG, fasting blood glucose; HDL-C, high density lipoprotein cholesterol; SUA, serum uric acid; TG, triglycerides; WC, waist circumference

^†^Cut-off values of baseline SUA quartiles in men: < 3.9 mg/dL, 3.9 to < 4.7 mg/dL, 4.7 to < 5.2 mg/dL, ≥ 5.2 mg/dL. Cut-off values of baseline SUA quartiles in women: < 3.2 mg/dL, 3.2 to < 3.7 mg/dL, 3.7 to < 4.3 mg/dL, ≥ 4.3 mg/dL

*ANOVA (for continuous variables) and chi-square test (for categorical variables) were used to compare basic characteristics across SUA quartile groups

^‡^Conversion factors for units: FBG in mg/dL to mmol/L, × 0.0555; TG in mg/dL to mmol/L, × 0.0113; HDL-C in mg/dL to mmol/L, × 0.0259

### Association between SUA levels and incident MetS

A total of 452 participants developed MetS during a follow-up of 4 years. In the fully adjusted model, the higher quartiles of baseline SUA were significantly associated with increased incident MetS. Compared to the lowest quartile of baseline SUA, the multivariable-adjusted OR (95% CI) for MetS in other three quartiles were 1.08 (0.77–1.50), 1.32 (0.95–1.82), and 1.55 (1.12–2.16) (Table 2). There was a linear trend in the odds of MetS across quartiles of baseline SUA (*P* for trend = 0.004). Each increment of 1 mg/dL in baseline SUA was associated with 19% higher odds of MetS (multivariable-adjusted OR 1.19; 95% CI 1.07–1.33). Participants with hyperuricemia had 47% higher odds of incident MetS than those without hyperuricemia (multivariable-adjusted OR 1.47; 95% CI 0.89–2.38), despite no statistical significance.

**Table 2:** ORs and 95% CIs for incident MetS according to SUA levels in the CHARLS

	MetS/total (%)	Model 1	Model 2	Model 3	*P* for trend*
*Quartiles of baseline SUA*					0.004
Quartile 1	84/941 (8.9)	Reference	Reference	Reference	
Quartile 2	93/948 (9.8)	1.11 (0.81–1.51)	1.10 (0.81–1.50)	1.08 (0.77–1.50)	
Quartile 3	117/944 (12.4)	1.44 (1.07–1.94)	1.40 (1.04–1.88)	1.32 (0.95–1.82)	
Quartile 4	158/946 (16.7)	2.05 (1.54–2.71)	1.99 (1.50–2.65)	1.55 (1.12–2.16)	
*Per 1 mg/dL increase*	–	1.20 (1.11–1.29)	1.30 (1.19–1.42)	1.19 (1.07–1.33)	
*Baseline hyperuricemia*					
No	423/3,645 (11.6)	Reference	Reference	Reference	
Yes	29/134 (21.6)	2.10 (1.38–3.21)	2.14(1.39–3.29)	1.47 (0.89–2.38)	
*Quartiles of absolute changes in SUA during follow–up*^†^					< 0.001
Quartile 1	93/945 (9.8)	Reference	Reference	Reference	
Quartile 2	101/944 (10.7)	1.10 (0.82–1.48)	1.05 (0.78–1.42)	1.23 (0.89–1.71)	
Quartile 3	107/945 (11.3)	1.17 (0.87–1.57)	1.13 (0.84–1.52)	1.39 (1.00–1.93)	
Quartile 4	151/945 (16.0)	1.74 (1.32–2.30)	1.77 (1.34–2.34)	1.89 (1.38–2.58)	
*Quartiles of percent changes in SUA during follow–up*^**‡**^					< 0.001
Quartile 1	88/944 (9.3)	Reference	Reference	Reference	
Quartile 2	106/945 (11.2)	1.23 (0.91–1.66)	1.19 (0.88–1.61)	1.32 (0.95–1.82)	
Quartile 3	113/946 (11.9)	1.32 (0.98–1.77)	1.29 (0.96–1.73)	1.51 (1.09–2.09)	
Quartile 4	145/944 (15.4)	1.77 (1.33–2.34)	1.76 (1.32–2.33)	2.18 (1.57–3.02)	

BMI, body mass index; BP, blood pressure; CHARLS, China Health and Retirement Longitudinal Study; CI, confidence interval; eGFR, estimated glomerular filtration rate; FBG, fasting blood glucose; HDL-C, high-density lipoprotein cholesterol; Mets, metabolic syndrome; OR, odd ratio; SUA, serum uric acid; TG, triglycerides; WC, waist circumference

Model 1: non–adjusted;

Model 2: adjusted for baseline age (continuous, years), gender (men and women), residence (urban and rural), education level (illiterate, primary school, middle school or above), marital status (married and unmarried), cigarette smoking (never, former, and current), alcohol drinking (never, former, and current);

Model 3: adjusted for baseline WC (cm), BMI (kg/m^2^), systolic BP (mmHg), FBG (mg/dL), TG (mg/dL), HDL-C (mg/dL), and eGFR (mL/min/1.73m^2^, all as continuous variables) in addition to covariates in Model 2. Baseline SUA (continuous, mg/dL) was additionally adjusted for in the analysis of quartiles of absolute changes (or percent changes) in SUA during follow-up

*P values for trend were estimated by modelling the serum uric acid using the median for each quartile

^†^Cut-off values of quartiles of absolute changes in SUA during follow-up: <  − 0.1 mg/dL, − 0.1 to < 0.5 mg/dL, 0.5 to < 1.0 mg/dL, ≥ 1.0 mg/dL

^‡^Cut-off values of quartiles of percent changes in SUA during follow-up: <  − 2.4%, − 2.4% to < 11.2%, 11.2% to < 26.8%, ≥ 26.8%

For three higher quartiles versus the lowest quartile of SUA changes (i.e., highest SUA decrease, <  − 0.1 mg/dL or <  − 2.4% change) over 4 years, the multivariable-adjusted OR (95% CI) for MetS were 1.23 (0.89–1.71), 1.39 (1.00–1.93), and 1.89 (1.38–2.58), respectively, when the absolute change was assessed as quartiles, and 1.32 (0.95–1.82), 1.51 (1.09–2.09), and 2.18 (1.57–3.02) respectively, when the percentage change was assessed as quartiles (Table 2). Linear trends were noted in these two sets of analyses (*P* for trend < 0.001).

There was no evidence of interactions of baseline SUA or absolute SUA changes with sex, age, or BMI regarding the associations with incident MetS (*P* for interaction ≥ 0.432 for all, Table 3). There were no apparent changes in the magnitude of associations when participants with hypertension or diabetes were excluded at baseline, compared to the main analyses (Additional file 1: Table S2).

**Table 3:** Adjusted ORs and 95% CIs across the quartiles of baseline SUA and SUA changes for incident MetS in major subgroups

Subgroups	*N*	Quartiles of baseline SUA^†^				*P* for interaction*	Quartiles of absolute changes in SUA during follow-up^†^				*P* for interaction*
		Quartile 1	Quartile 2	Quartile 3	Quartile 4		Quartile 1	Quartile 2	Quartile 3	Quartile 4	
*Sex*						0.767					0.432
Men	1783	Reference	1.09 (0.66–1.81)	1.20 (0.74–1.97)	1.53 (0.92–2.54)		Reference	1.17 (0.68–2.01)	1.76 (1.05–2.95)	2.06 (1.27–3.33)	
Women	1996	Reference	1.09 (0.70–1.71)	1.43 (0.93–2.20)	1.58 (1.02–2.45)		Reference	1.34 (0.88–2.03)	1.23 (0.81–1.91)	1.87 (1.22–2.85)	
*Age groups*						0.469					0.465
45–59 years	1945	Reference	0.96 (0.61–1.51)	1.58 (1.02–2.45)	1.65 (1.04–2.62)		Reference	0.95 (0.59–1.53)	1.27 (0.81–2.01)	1.65 (1.06–2.56)	
≥ 60 years	1834	Reference	1.24 (0.76–2.04)	1.15 (0.70–1.88)	1.47 (0.91–2.39)		Reference	1.63 (1.03–2.58)	1.46 (0.91–2.36)	2.15 (1.36–3.37)	
*BMI groups*						0.617					0.671
< 24 kg/m^2^	2628	Reference	0.94 (0.57–1.56)	1.21 (0.75–1.95)	1.58 (0.97–2.57)		Reference	0.99 (0.61–1.63)	1.39 (0.87–2.23)	1.85 (1.17–2.91)	
≥ 24 kg/m^2^	1151	Reference	1.14 (0.72–1.81)	1.28 (0.81–2.01)	1.51 (0.95–2.37)		Reference	1.59 (1.01–2.51)	1.45 (0.91–2.32)	1.86 (1.21–2.91)	

BMI, body mass index; BP, blood pressure; CI, confidence interval; eGFR, estimated glomerular filtration rate; FBG, fasting blood glucose; HDL-C, high-density lipoprotein cholesterol; Mets, metabolic syndrome; OR, odd ratio; SUA, serum uric acid; TG, triglycerides; WC, waist circumference

^†^Adjusted for baseline age (continuous, years), gender (men and women), residence (urban and rural), education level (illiterate, primary school, middle school or above), marital status (married and unmarried), cigarette smoking (never, former, and current), alcohol drinking (never, former, and current), WC (continuous, cm), BMI (continuous, kg/m^2^), systolic BP (continuous, mmHg), FBG (continuous, mg/dL), TG (continuous, mg/dL), and HDL-C (continuous, mg/dL), and eGFR (continuous, mL/min/1.73 m^2^). Baseline SUA (continuous, mg/dL) was additionally adjusted as covariate in the analysis of quartiles of absolute changes in SUA

*P values for interaction were estimated using the Wald test for the product term of the stratifying variable and SUA groups to the main model

**Fig. 1:**
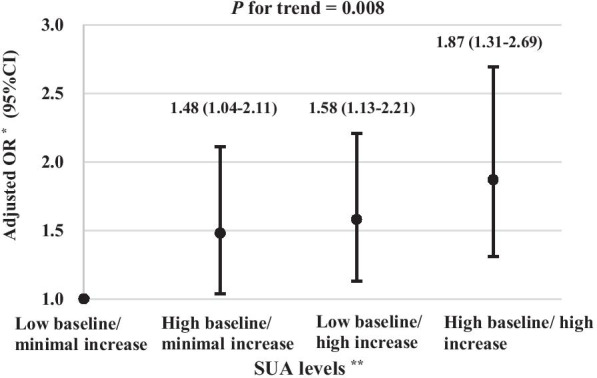
Association of combined baseline SUA and SUA changes with incident metabolic syndrome in the CHARLS. *Ab* BMI, body mass index; BP, blood pressure; CI, confidence interval; CHARLS, China Health and Retirement Longitudinal Study; eGFR, estimated glomerular filtration rate; FBG, fasting blood glucose; HDL-C, high-density lipoprotein cholesterol; OR, odd ratio; SUA, serum uric acid; TG, triglycerides; WC, waist circumference. *Adjusted for baseline age (continuous, years), gender (men and women), residence (urban and rural), education level (illiterate, primary school, middle school or above), marital status (married and unmarried), cigarette smoking (never, former, and current), alcohol drinking (never, former, and current), WC (continuous, cm), BMI (continuous, kg/m^2^), systolic BP (continuous, mmHg), FBG (continuous, mg/dL), TG (continuous, mg/dL), and HDL-C (continuous, mg/dL), and eGFR (continuous, mL/min/1.73 m^2^). **Baseline SUA levels and absolute SUA changes during the follow-up were dichotomized based on the median values (4.1 mg/dL and 0.5 mg/dL, respectively)

Compare to the low baseline/minimal increase category for SUA, the multivariable-adjusted OR (95% CI) for MetS were 1.48 (1.04–2.11), 1.58 (1.13–2.21), and 1.87 (1.31–2.69), respective, for the high baseline/minimal increase, low baseline/high increase, and high baseline/high increase (Fig. 1). There was a linear trend in the odds of MetS across the 4 categories (*P* = 0.008).

Baseline SUA were significantly associated with MetS components of elevated WC (multivariable OR [95% CI]: 1.41 [1.03–1.89] for Quartile 3 vs. 1), FBG (1.58 [1.04–2.41] for Quartile 4 vs. 1), and TG (1.72 [1.31–2.26] for Quartile 4 vs. 1), while absolute SUA changes were significantly associated with MetS components of elevated WC (1.51 [1.09–2.07] for Quartile 4 vs. 1), and TG (2.21 [1.71–2.86] for Quartile 4 vs. 1; Additional file 1: Table S3).

### Meta-analysis for associations between SUA levels and incident MetS

**Fig. 2:**
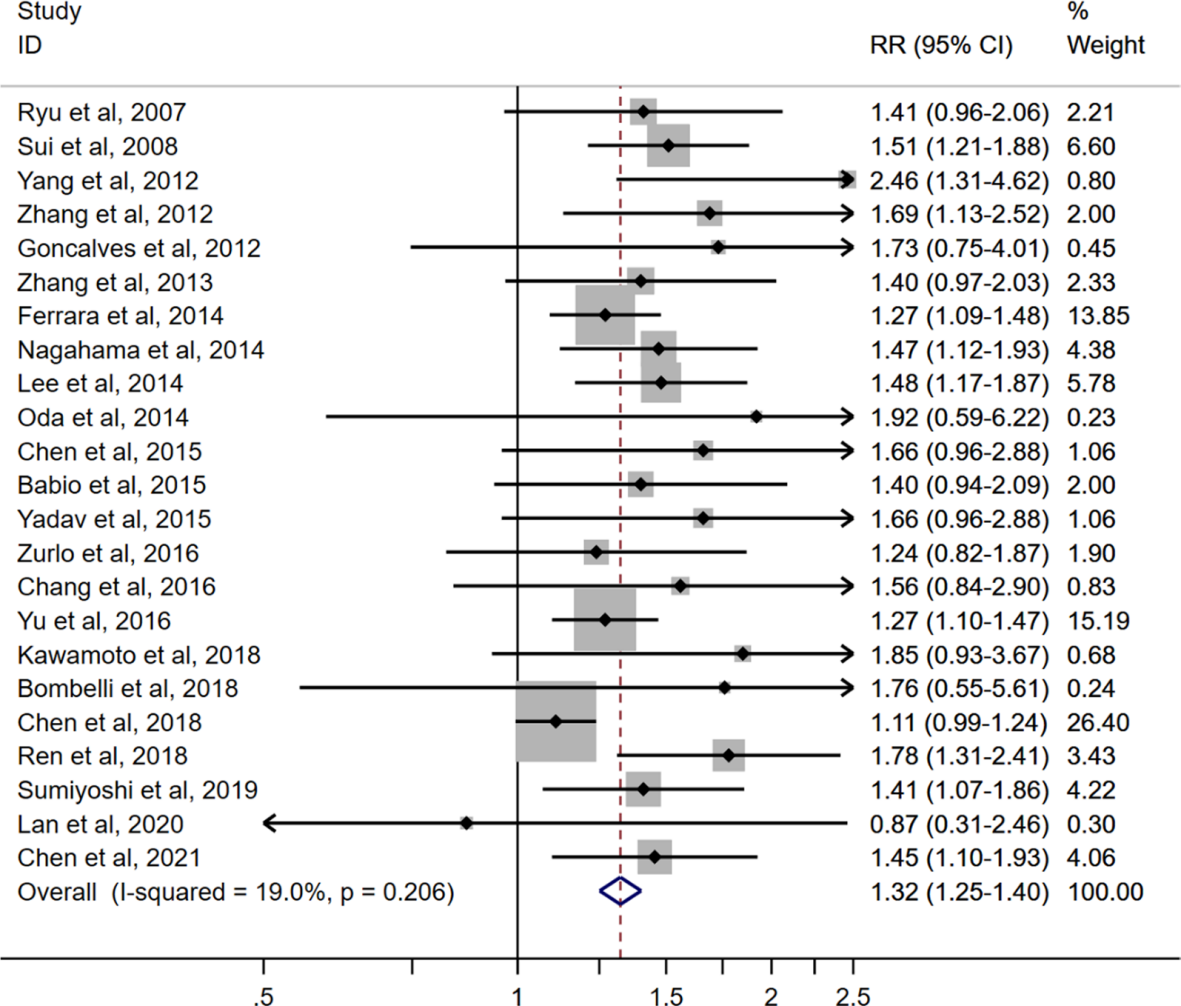
RRs for the highest versus lowest level of SUA in relation to incident metabolic syndrome. CI, confidence interval; RR, relative risk

A total of 3410 records were retrieved from three databases (679 in Embase, 831 in ISI Web of Science, and 1900 in PubMed), and 1087 duplicate records were excluded. The meta-analysis included a total of 24 cohort studies (including our study, Additional file 1: Figure S2) [7, 8, 19–39]. There were 140,913 participants and 17,575 incident cases of MetS with follow-up durations ranging from 2 to 11 years (Additional file 1: Table S4). Quality assessments of all included studies showed a Newcastle–Ottawa score of 7–8 (out of 9), which indicates overall good quality (Additional file 1: Table S5). 23 studies reported effect estimates for the highest versus lowest SUA group and 9 studies reported effect estimates for each 1 mg/dL increase in the SUA level. Pooled RR (95% CI) of incident MetS was 1.32 (1.25–1.40) for the highest versus lowest SUA group (Fig. 2) and 1.15 (1.09–1.21) for each 1 mg/dL increase in the SUA level (Additional file 1: Figure S3). The pooled RR was higher in the studies with median follow-up durations ≥ 4 years than those studies with follow-up < 4 years (pooled RR 1.38 vs. 1.29; *P* for heterogeneity = 0.014; Additional file 1: Table S6). No significant heterogeneity was noted between subgroups by region of studies, sex, or mean age (Additional file 1: Table S6). The funnel plot showed asymmetric distribution, and *P* values were < 0.001 and 0.509 for the Egger’s test and Begg’s test, indicating potential publication bias (Additional file 1: Figure S4).

## Discussion

In this large population-based cohort, both baseline SUA levels and increases in SUA during follow-up were positively associated with higher odds of MetS in middle-aged and elderly Chinese adults. The positive associations were consolidated by findings from a comprehensive meta-analysis of 24 cohort studies among 140,913 participants.

In our cohort study, participants in the highest SUA quartile had a 1.55-fold odds of incident MetS compared to those in the lowest quartile, and the association was linear across four quartiles. Consistently, four cohort studies in Chinese populations reported positive associations between SUA levels and incident MetS [7, 29, 36, 37], despite slight differences in the effect estimates. In particular, a large cohort study of 4988 participants showed that SUA levels were linearly associated with MetS over 9 years of follow-up in mainland China, and could be a strong and independent predictor of MetS in both men and women [29]. However, major limitations with these studies could include but were not limited to potential selection bias and residual confounding because study participants were enrolled solely from hospital settings and thus might have multiple comorbidities whose effects were difficult to adjust for in statistical analyses. In this regard, our study has been the only one that used a population-based cohort design in communities in mainland China, and our work improved the quality and generalizability of research findings on this topic in China. Consistent with our work, a few cohort studies in Korea and Japan also reported positive associations [25, 26, 28, 30, 32, 33, 35], of which several had a sample size over 10,000 [25, 32, 35]. Besides studies in East Asians, a study among 8429 men and 1260 women in the US found that there were 1.60- and 2.29-fold odds of MetS among participants in the upper tertile (≥ 6.5 mg/dL) versus the lower tertile (< 5.5 mg/dL) in a follow-up of 6.6 years [31]. Collectively, studies in different populations lent support to our finding of a positive association between baseline SUA and incident MetS in the Chinese population.

Despite strong evidence of positive association between baseline SUA and MetS, our original analyses on the link between SUA changes and incident MetS during follow-up were the first done among the Chinese population. Consistent with the finding for baseline SUA, longitudinal changes of SUA over 4 years were positively associated with odds of MetS, independent of baseline SUA. Similarly, a cohort study among 407 Japanese women reported that longitudinal increase across tertile of SUA levels over 11 years was associated with higher odds of MetS during [8], while another cohort study in 6083 Norwegian adults showed that each longitudinal increase of 59 μmol/L in SUA over 7 years was associated with 28% increased risk of incident MetS [27]. On the contrary, another cohort study among 13,057 Korean adults showed that percentage change of SUA was inversely associated with incident MetS during the 7-year follow-up [9]. Of note, it selectively enrolled participants from a medical center versus communities in other three studies, which might partially explain the discrepancies in research findings due to potential different profiles of participants.

In our meta-analysis of 24 cohort studies including 140,913 participants and 17,575 cases, a 32% increase in the risk of MetS was noted in the highest versus the lowest SUA group, which was slightly lower than the effect estimate reported in the meta-analysis in 2015 (pooled RR: 1.72) [6]. Compared to these previous analyses, our current meta-analysis comprised more than twice the original studies and study participants, which substantially improves the precision of effect estimates and statistical power for subgroup analyses. We found that the association between SUA and MetS was stronger in studies with longer follow-up durations, suggesting that high SUA predicts long-term risk of MetS. As most of the included studies in our meta-analysis and our current cohort study had a short average follow-up (for example, only four had a follow-up over 6 years) [8, 20, 27, 29], reverse causality could hardly be ruled out, i.e., the association might be explained as metabolic syndrome leading to high SUA. Thus, future large-scale population-based studies with long-term follow-ups are still warranted to dissect the effect of SUA on MetS. In addition, we observed consistent higher risk of MetS among participants with higher SUA across sex and age groups. Of note, accumulated evidence shows that elevated SUA levels might contribute to other cardiometabolic diseases such as diabetes, chronic kidney disease, and nonalcoholic fatty liver disease [24, 40], which supports the role of SUA in the development of MetS. Collectively, our original analyses in Chinese population and comprehensive meta-analysis across populations imply that monitoring the dynamic changes in SUA could be important for screening for high-risk individuals for MetS, especially for the middle-aged and elderly adults. From a clinical point of view, high SUA levels even below the limit for defining hyperuricemia and an increase in SUA over time might be indicative of potential higher risk of cardiometabolic diseases.

Although the underlying mechanisms for increased risk of MetS associated with higher SUA levels are not fully understood, there are several potential lines of evidence. First, elevated SUA levels have been reported to induce endothelial dysfunction and suppress endothelial nitric oxide bioavailability, leading to insulin resistance and oxidative stress that contribute to MetS [5, 41, 42]. Second, the rise in SUA is a strong predictor for the development of non-alcoholic fatty liver, which increases triglyceride accumulation as a component of MetS [43–45]. Third, experimental studies suggested that SUA may cause cardiovascular disorders such as hypertension by stimulating proliferation, angiotensin II production, and oxidative stress in vascular smooth muscle cell through renal and intracellular renin angiotensin system [46]. Fourth, diets that contain high fat and high fructose contribute to both SUA and features of metabolic syndrome [5], which implies that the association between SUA and metabolic syndrome may be partially explained by the confounding effect of unhealthy diets.

To the best of our knowledge, we conducted the first nation-wide population-based cohort study on this topic in China. It has strengths such as population-based design, large sample size, prospective design, structured questionnaire surveys for basic information, and standardized anthropometric and biochemical measurements. In addition, we updated the evidence through a comprehensive meta-analysis. However, some limitations should still be acknowledged. First, a large number of participants were lost to follow-up or did not provide sufficient information for our original analyses, and certain baseline characteristics such as age and BMI were different between included and exclude participants, which might lead to potential selection and information bias in our original work. However, our findings in the Chinese participants were corroborated by findings in the meta-analysis across populations. Second, the CHARLS did not collect detailed dietary or physical activity information so we could not adjust for the confounding effects of dietary factors such as intake of purine-rich foods, or physical activity. Third, due to data unavailability in the CHARLS, we could not exclude the participants who might use drugs known to influence SUA levels such as allopurinol, which may confound our estimates. Fourth, the current study among Chinese had a short follow-up so it may not capture sufficient cases for statistically powered subgroup analyses or rule out potential reverse causality.

## Conclusions

Higher baseline SUA and an increase in SUA over time were associated with the development of MetS among Chinese middle-aged and elderly adults. Such positive associations were supported by findings from an additional meta-analysis across multiple populations. Although future studies are still needed to explore the underlying mechanisms, SUA levels might need to be monitored closely for future risk of MetS in clinical practice.

## Supplementary Information

**Additional file 1: Table S1**. Comparison of baseline characteristics between participants included and excluded. **Table S2**. Association of SUA with incident MetS in participants without hypertension or diabetes at baseline. **Table S3**. Association of SUA with MetS components. **Table S4**. Basic information of studies included in the meta-analysis. **Table S5**. Newcastle-Ottawa quality assessments. **Table S6**. Subgroup analyses for the meta-analysis. **Figure S1**. Flowchart of participant eligibility. **Figure S2**. Flowchart of eligibility of studies for the meta-analysis. **Figure S3**. Association between each 1 mg/dL increase in the SUA and incident MetS. **Figure S4**. Funnel plot.

## Data Availability

The datasets used during the current study are available in the CHARLS official website, http://charls.pku.edu.cn/. All data, analytic methods, and study materials presented within this article are available from the corresponding author on reasonable request.

## Abbreviations

BMI: Body mass index
BP: Blood pressure
CHARLS: China Health and Retirement Longitudinal Study
CI: Confidence interval
eGFR: Estimated glomerular filtration rate
FBG: Fasting blood glucose
HR: Hazard ratio
HDL-C: High-density lipoprotein cholesterol
MetS: Metabolic syndrome
OR: Odd ratio
RR: Relative risk
SUA: Serum uric acid
TG: Triglycerides
WC: Waist circumference
