# Supplementary material for: Association between baseline and changes in serum uric acid and incident metabolic syndrome: a nation-wide cohort study and updated meta-analysis
Source: Nutr Metab (Lond). 2021 Jun 9;18:59. doi: 10.1186/s12986-021-00584-x (PMC8191036; doi:10.1186/s12986-021-00584-x)
Supplement: Supplementary file 1 — Additional file 1: Table S1. Comparison of baseline characteristics between participants included and excluded. Table S2. Association of SUA with incident MetS in participants without hypertension or diabetes at baseline. Table S3. Association of SUA with MetS components. Table S4. Basic information of studies included in the meta-analysis. Table S5. Newcastle-Ottawa quality assessments. Table S6. Subgroup analyses for the meta-analysis. Figure S1. Flowchart of participant eligibility. Figure S2. Flowchart of eligibility of studies for the meta-analysis. Figure S3. Association between each 1 mg/dL increase in the SUA and incident MetS. Figure S4. Funnel plot. [file 12986_2021_584_MOESM1_ESM.docx]

**Supplementary Materials**

**Table S1.** Comparison of baseline characteristics between participants included in and excluded from the CHARLS

| **Baseline characteristics ^†^** | **Participants included**  **(*n* =3,779)** | **Participants excluded**  **(*n* =3,182) ^‡^** | ***P* value** |
| --- | --- | --- | --- |
| Age, mean (SD), years | 59.5 (8.7) | 61.1 (10.4) | <0.001 |
| BMI, mean (SD), kg/m^2^ | 22.7 (3.3) | 22.3 (3.5) | <0.001 |
| SUA, mean (SD), mg/dL | 4.3 (1.2) | 4.4 (1.2) | <0.001 |
| Male, *n* (%) | 1783 (47.2) | 1556 (49.2) | 0.091 |
| Illiterate, *n* (%) | 1044 (27.6) | 991 (31.2) | 0.002 |
| Married, *n* (%) | 3368 (89.1) | 2730 (85.8) | <0.001 |
| Urban residence, *n* (%) | 1130 (29.9) | 1056 (33.2) | 0.003 |
| Current smokers, *n* (%) | 1195 (31.6) | 1069 (33.6) | 0.101 |
| Current alcohol drinkers, n (%) | 970 (25.7) | 852 (26.8) | 0.552 |
| None of Component of MetS, *n* (%) | 1073 (28.4) | 871 (27.4) | 0.584 |

**Abbreviations:** BMI, body mass index; CHARLS, China Health and Retirement Longitudinal Study; MetS, Metabolic syndrome; SD, standard deviation, SUA, serum uric acid.

^†^ Continuous variables were presented as mean (standard deviation), while categorical variables were presented as frequency (percentage). ANOVA (for continuous variables) and chi-square test (for categorical variables) were used to compare basic characteristics.

^‡^ Participants excluded (*n* =3,182) comprised those who lacked of information on MetS in 2015-2016 (*n* =2,435) or who lost to follow-up (*n* =747).

**Table S2**. Association of baseline SUA and SUA changes with incident MetS in participants without hypertension or diabetes at baseline (*N*=2366)

|  | **MetS / total (%)** | **Adjusted OR (95%CI)** | | | ***P* for trend** ^*^ |
| --- | --- | --- | --- | --- | --- |
|  |  | **Model 1** | **Model 2** | **Model 3** |  |
| **Quartiles of baseline SUA** |  |  |  |  | 0.013 |
| Quartile 1 | 37/625 (5.9) | Reference | Reference | Reference |  |
| Quartile 2 | 33/623 (5.3) | 0.89 (0.55–1.44) | 0.89 (0.55–1.45) | 0.81 (0.49–1.34) |  |
| Quartile 3 | 51/585 (8.7) | 1.52 (0.98–2.35) | 1.46 (0.94–2.28) | 1.21 (0.76–1.95) |  |
| Quartile 4 | 68/533 (12.8) | 2.32 (1.53–3.53) | 2.30 (1.51–3.51) | 1.59 (0.98–2.56) |  |
| **Per 1 mg/dL increase** | –– | 1.36 (1.21–1.53) | 1.46 (1.28–1.66) | 1.30 (1.11–1.53) |  |
| **Baseline hyperuricemia** |  |  |  |  |  |
| No | 175/2300 (7.6) | Reference | Reference | Reference |  |
| Yes | 14/66 (21.2) | 3.27 (1.78–6.02) | 3.40 (1.83–6.33) | 1.91 (0.93–3.93) |  |
| **Quartiles of absolute changes in SUA during follow–up ^†^** |  |  |  |  | 0.001 |
| Quartile 1 | 31/571 (5.4) | Reference | Reference | Reference |  |
| Quartile 2 | 51/629 (8.1) | 1.54 (0.97–2.44) | 1.45 (0.91–2.31) | 1.40 (0.86–2.30) |  |
| Quartile 3 | 45/605 (7.4) | 1.40 (0.87–2.25) | 1.34 (0.84–2.16) | 1.30 (0.78–2.15) |  |
| Quartile 4 | 62/561 (11.1) | 2.16 (1.38–3.39) | 2.15 (1.37–3.38) | 1.94 (1.20–3.16) |  |
| **Quartiles of percent changes in SUA during follow–up ^‡^** |  |  |  |  | 0.001 |
| Quartile 1 | 29/571 (5.1) | Reference | Reference | Reference |  |
| Quartile 2 | 51/618 (8.3) | 1.68 (1.05–2.69) | 1.58 (0.98–2.54) | 1.60 (0.96–2.67) |  |
| Quartile 3 | 51/589 (8.7) | 1.77 (1.11–2.84) | 1.71 (1.07–2.75) | 2.08 (1.24–3.49) |  |
| Quartile 4 | 58/588 (9.9) | 2.05 (1.29–3.24) | 2.02 (1.27–3.22) | 2.63 (1.55–4.46) |  |

**Abbreviations**: BMI, body mass index; BP, blood pressure; CI, confidence interval; eGFR, estimated glomerular filtration rate; FBG, fasting blood glucose; HDL-C, high-density lipoprotein cholesterol; OR, odd ratio; SUA, serum uric acid; TG, triglycerides; WC, waist circumference.

Model 1: non–adjusted;

Model 2: adjusted for baseline age (continuous, years), gender (men and women), residence (urban and rural), education level (illiterate, primary school, middle school or above), marital status (married and unmarried), cigarette smoking (never, former, and current), alcohol drinking (never, former, and current);

Model 3: adjusted for baseline WC (cm), BMI (kg/m^2^), systolic BP (mmHg), FBG (mg/dL), TG (mg/dL), HDL-C (mg/dL), and eGFR (mL/min/1.73m^2^, all as continuous variables) in addition to covariates in Model 2. Baseline SUA (continuous, mg/dL) was additionally adjusted for as a covariate in the analysis of quartiles of absolute changes (or percent changes) in SUA during follow–up.

**^*^** *P* values for trend were estimated by modelling the serum uric acid using the median for each quartile.

**^†^** Cut-off values of quartiles of absolute changes in SUA during follow–up: < –0.1 mg/dL, –0.1 to < 0.5 mg/dL, 0.5 to < 1.0 mg/dL, ≥ 1.0 mg/dL.

**^‡^** Cut-off values of quartiles of percent changes in SUA during follow–up: < –2.4%, –2.4% to < 11.2%, 11.2% to < 26.8%, ≥ 26.8%.

**Table S3.** Association of baseline SUA and SUA changes with MetS components during follow–up

|  | **Incident case /**  **sample size (rate: %)** | **Quartiles of baseline SUA ^†^** | | | | **Quartiles of absolute changes in SUA** **during follow–up ^†^** | | | |
| --- | --- | --- | --- | --- | --- | --- | --- | --- | --- |
|  |  | **Quartile 1** | **Quartile 2** | **Quartile 3** | **Quartile 4** | **Quartile 1** | **Quartile 2** | **Quartile 3** | **Quartile 4** |
| **Elevated WC** |  |  |  |  |  |  |  |  |  |
| **Incidents / total (%)** | 504/2692 (18.7) | 119/719 (16.6) | 104/683 (15.2) | 149/667 (22.3) | 132/623 (21.2) | 120/715 (16.8) | 118/648 (18.2) | 124/679 (18.3) | 142/650 (21.8) |
| **Adjusted OR (95%CI)** | –– | Reference | 0.93 (0.67–1.28) | **1.41 (1.03–1.89)** | 1.24 (0.89–1.74) | Reference | 1.07 (0.77–1.47) | 1.18 (0.86–1.63) | **1.51 (1.09–2.07)** |
| **Elevated FBG** |  |  |  |  |  |  |  |  |  |
| **Incidents / total (%)** | 218/3209 (6.8) | 47/791 (5.9) | 51/800 (6.4) | 44/810 (5.4) | 76/808 (9.4) | 59/792 (7.4) | 46/796 (5.8) | 45/808 (5.6) | 68/813 (8.4) |
| **Adjusted OR (95%CI)** | –– | Reference | 1.12 (0.74–1.71) | 0.91 (0.58–1.39) | **1.58 (1.04–2.41)** | Reference | 0.86 (0.57–1.31) | 0.84 (0.55–1.28) | 1.16 (0.78–1.71) |
| **Elevated TG** |  |  |  |  |  |  |  |  |  |
| **Incidents / total (%)** | 598/3387 (17.7) | 118/868 (13.6) | 154/870 (17.7) | 159/837 (19.0) | 167/812 (20.6) | 116/838 (13.8) | 133/850 (15.6) | 152/855 (17.8) | 197/844 (23.3) |
| **Adjusted OR (95%CI)** | –– | Reference | 1.29 (0.99–1.69) | **1.47 (1.13–1.91)** | **1.72 (1.31–2.26)** | Reference | 1.18 (0.91–1.54) | **1.54 (1.18–2.01)** | **2.21 (1.71–2.86)** |
| **Reduced HDL-C** |  |  |  |  |  |  |  |  |  |
| **Incidents / total (%)** | 202/3377 (6.0) | 47/824 (5.7) | 46/859 (5.4) | 53/848 (6.3) | 56/846 (6.6) | 60/860 (7.0) | 50/867 (5.8) | 41/842 (4.9) | 51/808 (6.3) |
| **Adjusted OR (95%CI)** | –– | Reference | 0.91 (0.59–1.39) | 1.07 (0.71–1.64) | 1.06 (0.68–1.65) | Reference | 0.87 (0.58–1.31) | 0.72 (0.47–1.11) | 0.83 (0.55–1.25) |
| **Elevated BP** |  |  |  |  |  |  |  |  |  |
| **Incidents / total (%)** | 589/2256 (26.1) | 148/605 (24.5) | 145/607 (23.9) | 147/549 (26.8) | 149/495 (30.1) | 159/532 (29.9) | 158/608 (26.0) | 135/581 (23.2) | 137/535 (25.6) |
| **Adjusted OR (95%CI)** | –– | Reference | 0.94 (0.72–1.23) | 1.05 (0.79–1.39) | 1.14 (0.84–1.53) | Reference | 0.89 (0.67–1.17) | 0.75 (0.57–1.00) | 0.84 (0.63–1.12) |

**Abbreviations:** BMI, body mass index; BP, blood pressure; CI, confidence interval; FBG, fasting blood glucose; HDL-C, high-density lipoprotein cholesterol; MetS, metabolic syndrome; OR, odd ratio; SUA, serum uric acid; TG, triglycerides; WC, waist circumference.

^†^ Adjusted for baseline age (continuous, years), gender (men and women), residence (urban and rural), education level (illiterate, primary school, middle school or above), marital status (married and unmarried), cigarette smoking (never, former, and current), alcohol drinking (never, former, and current), WC (continuous, cm), BMI (continuous, kg/m^2^), systolic BP (continuous, mmHg), FBG (continuous, mg/dL), TG (continuous, mg/dL), and HDL–C (continuous, mg/dL), and eGFR (continuous, mL/min/1.73m^2^). Baseline SUA (continuous, mg/dL) was additionally adjusted for as a covariate in the analysis of quartiles of absolute changes in SUA.

**Table S4.** Basic information of studies included in the meta–analysis.

| **First author** | **Year** | **Country/**  **region** | **Mean Age,**  **Years** | **Sex (female /male)** | **Case/**  **sample size** | **Diagnostic**  **criteria** | **Follow–up, years** | **Type of effect estimate** | **Effect size (95% CI)** |
| --- | --- | --- | --- | --- | --- | --- | --- | --- | --- |
| Ryu S[1] | 2007 | Korea | 33.2 | 0/4779 | 708/4779 | NCEP ATP III | 2.10 | IRR | 1.41 (1.08–1.84) |
| Sui X[2] | 2008 | United  States | 43.7 | 1260/8429 | 1164/9689 | NCEP ATP III | 5.70 | OR | Male: 1.60 (1.34–1.91)  Female: 2.29 (1.00–5.27) |
| Yang T[3] | 2012 | China (Taiwan) | 40.8 | 2109/1748 | 476/3857 | UCS | 5.41 | HR | All: 2.46 (1.91–3.17)  Male: 1.38 (0.86–2.66)  Female: 3.18 (2.20–4.60) |
| Zhang Q[4] | 2012 | China (mainland) | 46.5 | 0/2222 | NA/2222 | CDS | 6.00 | OR | 1.69 (1.34–2.14) |
| Goncalves JP[5] | 2012 | Portugal | 49.8 | NA | NA/1054 | Joint Interim criteria | 5.00 | IRR | 1.73 (1.08–2.76)  1.22 (1.05–1.42) ^†^ |
| Zhang ML[6] | 2013 | China (mainland) | 49.5 | 4442/2957 | 1190/7399 | IDF | 3.00 | HR | Male: 1.29 (1.01–1.67)  Female: 1.62 (1.24–2.11) |
| Ferrara LA[7] | 2014 | United States | 32.7 | 890/609 | 454/1499 | NCEP ATP III | 4.00 | OR | 1.44 (1.14–1.81)  1.11 (0.99–1.25) ^†^ |
| Nagahama K[8] | 2014 | Japan | 48.7 | 2792/3144 | 944/5936 | Japanese | 4.00 | OR | Male: 1.50 (1.30–1.80)  Female: 2.00 (1.30–3.00) |
| Lee JK[9] | 2014 | Korea | 44.6 | 0/14906 | 2428/14906 | IDF | 5.00 | HR | 1.48 (1.26–1.73) |
| Oda E[10] | 2014 | Japan | 51.6 | 953/1606 | 248/2559 | NCEP ATP III | 2.50 | HR | Male: 1.90 (1.38–2.62)  Female: 2.09 (1.04–4.19)  Male: 1.28 (1.10–1.50) ^†^  Female: 1.35 (1.04–1.76) ^†^ |
| Chen D[11] | 2015 | China (mainland) | 39.4 | 0/2575 | 348/2575 | NCEP ATP III | 3.00 | HR | Male: 1.66 (1.20–2.30) |
| Babio N[12] | 2015 | Spain | 76.1 | NA | 753/1511 | HIDF AHLB | 3.80 | HR | All: 1.40 (1.10–1.90)  Male: 1.30 (1.00–1.80)  Female: 1.80 (1.30–2.50) |
| Yadav D[13] | 2015 | Korea | 54.4 | 929/661 | 261/1590 | HIDF AHLB | 2.60 | OR | Male: 1.89 (1.03–3.47)  Female: 1.93 (1.11–3.40) |
| Zurlo A[14] | 2016 | [Italy](javascript:;) | 74.7 | 610/518 | 496/1128 | HIDF AHLB | 4.40 | HR | All: 1.24 (0.90–1.72)  Male: 0.62 (0.36–1.07)  Female: 1.58 (1.03–2.40)  All: 1.00 (0.90–1.11) ^†^  Male: 1.00 (0.83–1.22) ^†^  Female: 1.00 (0.87–1.14) ^†^ |
| Chang JB[15] | 2016 | China (Taiwan) | 70.8 | 4752/6405 | NA/11157 | HIDF AHLB | 4.30 | HR | Male:  65–74 years: 1.57 (1.26–1.98)  75–84 years: 1.90 (1.08–3.34)  Female:  65–74 years: 1.58 (1.25–2.00)  75–84 years: 1.17 (0.56–2.43) |
| Norvik JV[16] | 2016 | Norway | 60 | 3087 /2996 | 611/6083 | NCEP ATP III | 7.00 | OR | 1.29 (1.18–1.41) ^†^ |
| Yu TY[17] | 2016 | Korea | 50.8 | 5727/8715 | 4215/14442 | IDF | 4.43 | HR | Male: 1.25 (1.12–1.39)  Female: 1.32 (1.11–1.57)  Male: 1.07 (1.03–1.11) ^†^  Female: 1.11 (1.01–1.22) ^†^ |
| Kawamoto R[18] | 2018 | Japan | 71.0 | 324/0 | 107/324 | NCEP ATP III | 11.00 | OR | Female: 3.20 (1.62–6.35) |
| Bombelli M[19] | 2018 | Italy | 47.4 | 602/590 | 108/1192 | NCEP ATP III | 10.00 | RR | 1.76 (0.95–3.27)  1.10 (0.90–1.35) ^†^ |
| Chen YY[20] | 2018 | China (Taiwan) | 30.4 | NA | NA/20247 | NCEP ATP III | 2.00 | HR | Male:  <30 years: 1.06 (0.90–1.25)  30–40 years: 1.12 (1.01–1.25)  >40 years: 2.99 (1.34–6.64)  Female:  <30 years: 1.22 (0.96–1.55)  30–40 years: 1.24 (0.85–1.79)  >40 years: 1.12 (0.86–1.46) |
| Ren P[21] | 2018 | China (mainland) | 41.2 | 1625/3363 | 1192/4988 | Chinese | 9.00 | HR | 1.78 (1.50–2.11) |
| Sumiyoshi H[22] | 2019 | Japan | 48.9 | 5931/8862 | 1031/14793 | Japanese | 3.00 | HR | All: 1.41 (1.16–1.71)  Male: 1.08 (1.02–1.15)  Female: 1.31 (1.06–1.61)  All: 1.10 (1.04–1.17) ^†^ |
| Lan Q[23] | 2020 | China (mainland) | 68.0 | 1825/1379 | 389/3204 | American  Heart Association  Guidelines | 4.00 | HR | Male: 1.60 (0.93–2.77)  Female: 0.56 (0.31–1.01) |
| Current study | 2020 | China (mainland) | 59.5 | 1996/1783 | 452/3779 | Chinese | 4.00 | OR | All: 1.55 (1.12–2.16)  Male: 1.53 (0.92–2.54)  Female: 1.58 (1.02–2.45)  All: 1.19 (1.07–1.33) ^†^ |

**Abbreviations**: CDS, Diabetes Branch of the Chinese Medical Association; HIDF AHLB, Updated Harmonized Criteria of the International Diabetes Federation and the American Heart Association/National Heart, Lung, and Blood Institute; HR, hazard ratio; IDF, The International Diabetes Federation criteria; IRR, incidence rate ratio; NCEP ATP III, National Cholesterol Education Program Adult Treatment Panel III criteria; RR, relative risk; OR, odds ratio; UCS, Unified criteria set by several major organizations (International Diabetes Federation Task Force on Epidemiology and Prevention; National Heart, Lung, and Blood Institute; American Heart Association; Word Heart Federation; International Atherosclerosis Society; and International Association for the Study of Obesity).

^†^ Effect size were calculated using SUA as a continuous variable in relation to new-onset MetS.

**Table S5.** Newcastle–Ottawa quality assessments for studies included in our meta–analysis.

| **Study (first author, year)** | **Selection** | | | | **Comparability** | **Outcome** | | | **Total score** |
| --- | --- | --- | --- | --- | --- | --- | --- | --- | --- |
|  | Representativeness of the exposed cohort | Selection of the non–exposed cohort | Ascertainment of exposure | Demonstration that outcome of interest was not present at start of study | Comparability of cohorts on the basis of the design or analysis | Assessment of outcome | Was follow–up long enough for outcomes to occur | Adequacy of follow up of cohorts |  |
| Ryu S[1] | 0 | 1 | 1 | 1 | 2 | 1 | 1 | 0 | 7 |
| Sui X[2] | 1 | 1 | 1 | 1 | 2 | 1 | 1 | 0 | 8 |
| Yang T[3] | 1 | 1 | 1 | 1 | 2 | 1 | 1 | 0 | 8 |
| Zhang Q[4] | 1 | 1 | 1 | 1 | 2 | 1 | 1 | 0 | 8 |
| Goncalves JP[5] | 1 | 1 | 1 | 1 | 2 | 1 | 1 | 0 | 8 |
| Zhang ML[6] | 1 | 1 | 1 | 1 | 2 | 1 | 1 | 0 | 8 |
| Ferrara LA[7] | 1 | 1 | 1 | 1 | 2 | 1 | 1 | 0 | 8 |
| Nagahama K[8] | 1 | 1 | 1 | 1 | 2 | 1 | 1 | 0 | 8 |
| Lee JK[9] | 1 | 1 | 1 | 1 | 2 | 1 | 1 | 0 | 8 |
| Oda E[10] | 1 | 1 | 1 | 1 | 2 | 1 | 1 | 0 | 8 |
| Chen D[11] | 1 | 1 | 1 | 1 | 2 | 1 | 1 | 0 | 8 |
| Babio N[12] | 1 | 1 | 1 | 1 | 2 | 1 | 1 | 0 | 8 |
| Yadav D[13] | 1 | 1 | 1 | 1 | 2 | 1 | 1 | 0 | 8 |
| Zurlo A[14] | 1 | 1 | 1 | 1 | 2 | 1 | 1 | 0 | 8 |
| Chang JB[15] | 1 | 1 | 1 | 1 | 2 | 1 | 1 | 0 | 8 |
| Norvik JV[16] | 0 | 1 | 1 | 1 | 2 | 1 | 1 | 1 | 8 |
| Yu TY[17] | 1 | 1 | 1 | 1 | 2 | 1 | 1 | 0 | 8 |
| Kawamoto R[18] | 1 | 1 | 1 | 1 | 2 | 1 | 1 | 0 | 8 |
| Bombelli M[19] | 1 | 1 | 1 | 1 | 2 | 1 | 1 | 0 | 8 |
| Chen YY[20] | 1 | 1 | 1 | 1 | 1 | 1 | 1 | 0 | 7 |
| Ren P[21] | 1 | 1 | 1 | 1 | 2 | 1 | 1 | 0 | 8 |
| Sumiyoshi H[22] | 1 | 1 | 1 | 1 | 2 | 1 | 1 | 0 | 8 |
| Lan Q[23] | 1 | 1 | 1 | 0 | 2 | 1 | 1 | 0 | 7 |

**Table S6.** Subgroup analyses for the meta–analysis

| **Subgroups** | **Number of cohorts** | **RR (95% CI)** | **I^2^ (%)** | ***P* for heterogeneity** |
| --- | --- | --- | --- | --- |
| **Region of studies** |  |  |  | 0.818 |
| Asian | 17 | 1.32 (1.23–1.41) | 35.1 |  |
| America | 2 | 1.34 (1.19–1.52) | 37.5 |  |
| Europe | 4 | 1.38 (1.06–1.79) | 0.0 |  |
| **Sex** |  |  |  | 0.497 |
| Men | 15 | 1.26 (1.13–1.41) | 69.4 |  |
| Women | 15 | 1.32 (1.21–1.46) | 62.4 |  |
| **Mean age (years)** |  |  |  | 0.154 |
| <45 | 7 | 1.41 (1.20–1.67) | 65.7 |  |
| 45–65 | 12 | 1.41 (1.29–1.54) | 0.0 |  |
| ≥66 | 4 | 1.32 (1.03–1.70) | 0.0 |  |
| **Median follow–up (years)** |  |  |  | 0.014 |
| <4 | 8 | 1.29 (1.14–1.46) | 16.8 |  |
| ≥4 | 15 | 1.38 (1.28–1.48) | 0.0 |  |

**Abbreviations:** RR, relative risk; CI, confidence interval.

.

**F****igure S1.** Flowchart of participant eligibility in the China Health and Retirement Longitudinal Study.

The China Health and Retirement Longitudinal Study

(2011-2012; *N* = 17,708)

Included for analyses

(n = 3,779)

Lack of information on MetS in 2015-2016 (*n* = 2,435)

Availability of questionnaires and blood samples (*n* = 10,131)

(n=)

Lack of information on MetS at baseline (*n* = 456)

Lack of information on SUA at baseline (*n* = 163)

Loss to follow up in

2015-2016 (*n* = 747)

With MetS at baseline

(*n* = 2,551)

**Abbreviations:** MetS, metabolic syndrome; SUA, serum uric acid.

23 cohort studies included

2,269 records were excluded based on titles

1,087 duplicates were excluded

31 studies were excluded:

- 6 reviews
- 18 cross–section studies
- 2 studies not on SUA as the exposure
- 2 studies in adolescents
- 2 studies only on the change of SUA
- 1 study not excluding MetS at baseline

2,323 relevant records included

54 studies for further screening

3,410 records included:

- 679 in Embase
- 831 in ISI Web of Science
- 1,900 in PubMed

**Figure S2.** Flowchart of eligibility of studies for the meta–analysis.

**Abbreviations:** MetS, metabolic syndrome; SUA, serum uric acid.


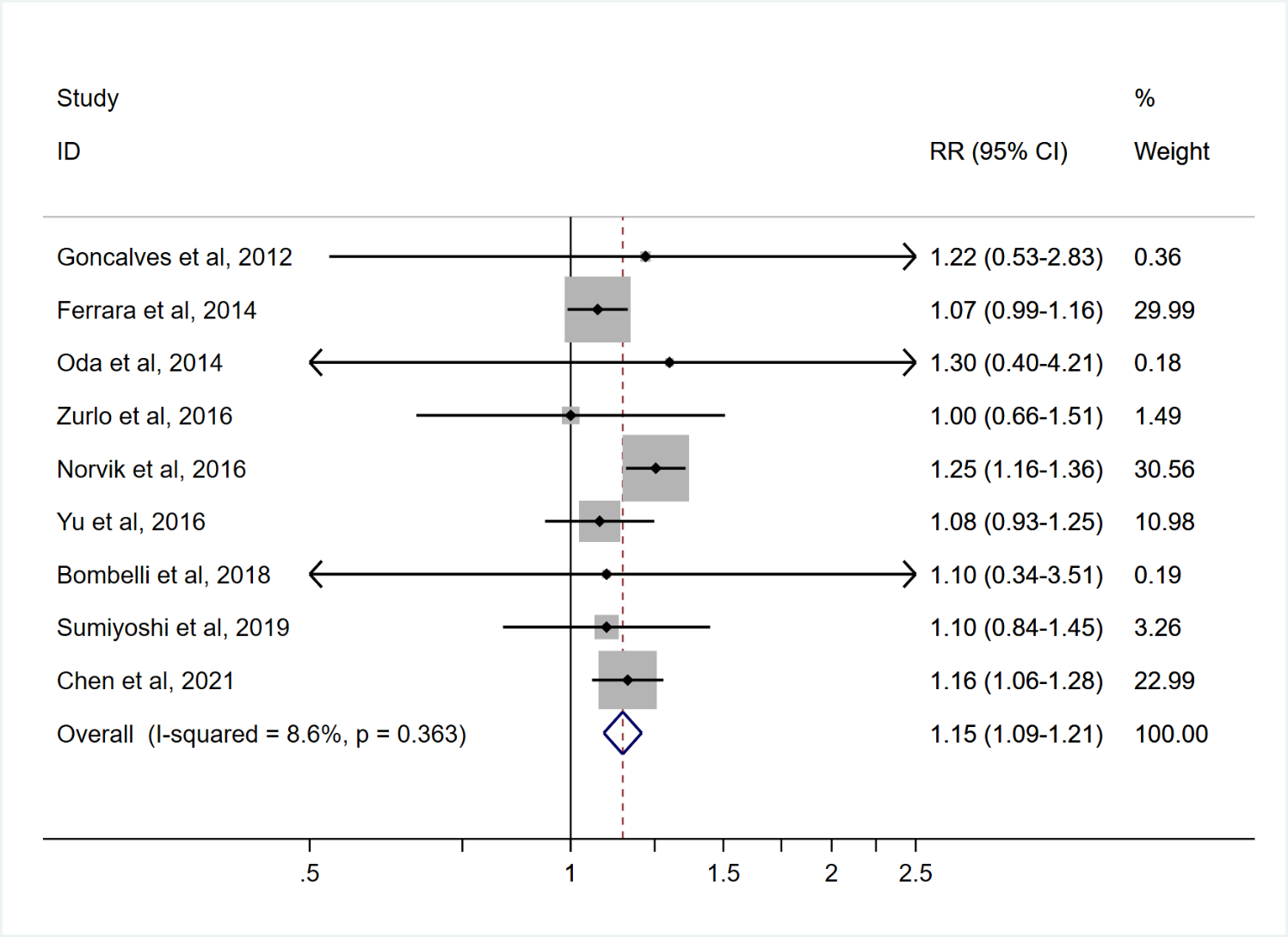


**Figure S3.** Association between each 1 mg/dL increase in the SUA level and incident metabolic syndrome.

**Abbreviations:** CI, confidence interval; RR, relative risk; SUA, serum uric acid


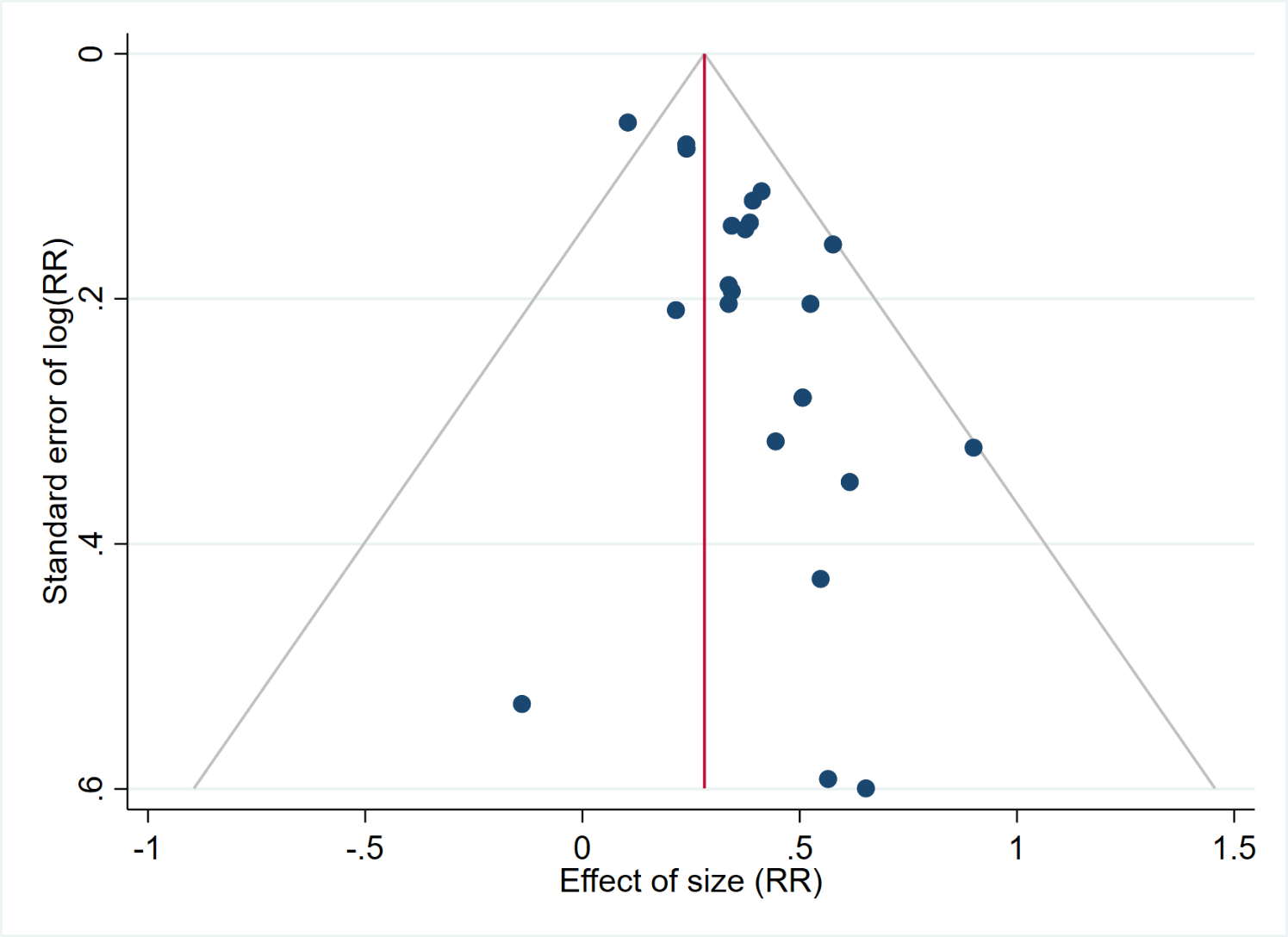


**Figure S4.** Funnel plot of meta–analysis of included studies.

**Abbreviations:** RR, relative risk.

The funnel plot showed asymmetric distribution, and *P* values were < 0.001 and 0.509 for the Egger’s test and Begg’s test, indicating potential publication bias.

**References**

1. Ryu S, Song J, Choi BY, Lee SJ, Kim WS, Chang Y, Kim DI, Suh BS, Sung KC. Incidence and risk factors for metabolic syndrome in Korean male workers, ages 30 to 39. Ann Epidemiol. 2007; 17:245-252.

2. Sui X, Church TS, Meriwether RA, Lobelo F, Blair SN. Uric acid and the development of metabolic syndrome in women and men. Metabolism-Clinical and Experimental. 2008; 57:845-852.

3. Yang T, Chu CH, Bai CH, You SL, Chou YC, Chou WY, Chien KL, Hwang LC, Su TC, Tseng CH, et al. Uric acid level as a risk marker for metabolic syndrome: A Chinese cohort study. Atherosclerosis. 2012; 220:525-531.

4. Zhang Q, Zhang C, Song X, Lin H, Zhang D, Meng W, Zhang Y, Zhu Z, Tang F, Liu L, et al. A longitudinal cohort based association study between uric acid level and metabolic syndrome in Chinese Han urban male population. BMC Public Health. 2012; 12:419.

5. Goncalves JP, Oliveira A, Severo M, Santos AC, Lopes C. Cross-sectional and longitudinal associations between serum uric acid and metabolic syndrome. Endocrine. 2012; 41:450-457.

6. Zhang M, Gao Y, Wang X, Chang H, Huang G. Serum uric acid and appropriate cutoff value for prediction of metabolic syndrome among Chinese adults. Journal of Clinical Biochemistry and Nutrition. 2013; 52:38-42.

7. Ferrara LA, Wang H, Umans JG, Franceschini N, Jolly S, Lee ET, Yeh J, Devereux RB, Howard BV, de Simone G. Serum uric acid does not predict incident metabolic syndrome in a population with high prevalence of obesity. Nutrition Metabolism and Cardiovascular Diseases. 2014; 24:1360-1364.

8. Nagahama K, Inoue T, Kohagura K, Ishihara A, Kinjo K, Ohya Y. Hyperuricemia predicts future metabolic syndrome: a 4-year follow-up study of a large screened cohort in Okinawa, Japan. Hypertension Research. 2014; 37:232-238.

9. Lee JK, Ryoo JH, Choi JM, Park SK. Serum uric acid level and the incidence of metabolic syndrome in middle-aged Korean men: a 5-year follow-up study. Journal of preventive medicine and public health. 2014; 47:317-326.

10. Oda E. Serum uric acid is an independent predictor of metabolic syndrome in a Japanese health screening population. Heart and Vessels. 2014; 29:496-503.

11. Chen DN, Zhang HY, Gao Y, Lu Z, Yao ZT, Jiang YH, Lin XG, Wu CL, Yang XB, Tan AH, et al. Cross-sectional and longitudinal associations between serum uric acid and metabolic syndrome: Results from Fangchenggang Area Male Health and Examination Survey in China. Clinica Chimica Acta. 2015; 446:226-230.

12. Babio N, Martinez-Gonzalez MA, Estruch R, Waernberg J, Recondo J, Ortega-Calvo M, Serra-Majem L, Corella D, Fito M, Ros E, et al. Associations between serum uric acid concentrations and metabolic syndrome and its components in the PREDIMED study. Nutrition Metabolism and Cardiovascular Diseases. 2015; 25:173-180.

13. Yadav D, Lee ES, Kim HM, Choi E, Lee EY, Lim JS, Ahn SV, Koh SB, Chung CH. Prospective study of serum uric acid levels and incident metabolic syndrome in a Korean rural cohort. Atherosclerosis. 2015; 241:271-277.

14. Zurlo A, Veronese N, Giantin V, Maselli M, Zambon S, Maggi S, Musacchio E, Toffanello ED, Sartori L, Perissinotto E, et al. High serum uric acid levels increase the risk of metabolic syndrome in elderly women: The PRO.V.A study. Nutrition Metabolism and Cardiovascular Diseases. 2016; 26:27-35.

15. Chang JB, Chen YL, Hung YJ, Hsieh CH, Lee CH, Pei D, Lin JD, Wu CZ, Liang YJ, Lin CM. The role of uric acid for predicting future metabolic syndrome and type 2 diabetes in older people. Journal of Nutrition, Health & Aging. 2016; 21:329-335.

16. Norvik JV, Storhaug HM, Ytrehus K, Jenssen TG, Zykova SN, Eriksen BO, Solbu MD. Overweight modifies the longitudinal association between uric acid and some components of the metabolic syndrome: The Tromso Study. BMC Cardiovascular Disorders. 2016; 16:85.

17. Yu TY, Jee JH, Bae JC, Jin SM, Baek JH, Lee MK, Kim JH. Serum uric acid: A strong and independent predictor of metabolic syndrome after adjusting for body composition. Metabolism-Clinical and Experimental. 2016; 65:432-440.

18. Kawamoto R, Ninomiya D, Kasai Y, Senzaki K, Kusunoki T, Ohtsuka N, Kumagi T. Baseline and changes in serum uric acid independently predict 11-year incidence of metabolic syndrome among community-dwelling women. Journal of Endocrinological Investigation. 2018; 41:959-968.

19. Bombelli M, Quarti-Trevano F, Tadic M, Facchetti R, Cuspidi C, Mancia G, Grassi G. Uric acid and risk of new-onset metabolic syndrome, impaired fasting glucose and diabetes mellitus in a general Italian population: data from the Pressioni Arteriose Monitorate E Loro Associazioni study. Journal of Hypertension. 2018; 36:1492-1498.

20. Chen Y, Kao T, Yang H, Chou C, Wu C, Lai C, Sun Y, Wang C, Chen W. The association of uric acid with the risk of metabolic syndrome, arterial hypertension or diabetes in young subjects- An observational study. Clinica Chimica Acta. 2018; 478:68-73.

21. Ren P, Gao MN. Association between metabolic syndrome and the serum uric acid: A cohort Study. Clinical Laboratory. 2018; 64:719-726.

22. Sumiyoshi H, Ohyama Y, Imai K, Kurabayashi M, Saito Y, Nakamura T. Association of uric acid with incident metabolic syndrome in a Japanese general population. International Heart Journal. 2019; 60:830-835.

23. Lan Q, Wu H, Zhou XH, Zheng L, Lin F, Meng QS, Xi XL, Yue AX, Buys N, Sun J, et al. Predictive value of uric acid regarding cardiometabolic disease in a community-dwelling older population in Shanghai: A cohort study. Frontiers in Medicine. 2020; 7:24.

.

**STROBE Statement checklist for current cohort study**

|  | Item NO. | Recommendation | Page NO. |
| --- | --- | --- | --- |
| **Title and abstract** | 1 | (*a*) Indicate the study’s design with a commonly used term in the title or the abstract | 3 |
|  |  | (*b*) Provide in the abstract an informative and balanced summary of what was done and what was found | 3 |
| Introduction | | |  |
| Background/rationale | 2 | Explain the scientific background and rationale for the investigation being reported | 4 |
| Objectives | 3 | State specific objectives, including any prespecified hypotheses | 5 |
| Methods | | |  |
| Study design | 4 | Present key elements of study design early in the paper | 5 |
| Setting | 5 | Describe the setting, locations, and relevant dates, including periods of recruitment, exposure, follow–up, and data collection | 5 |
| Participants | 6 | (*a*) Give the eligibility criteria, and the sources and methods of selection of participants. Describe methods of follow–up | 6 |
|  |  | (*b*) For matched studies, give matching criteria and number of exposed and unexposed | NA |
| Variables | 7 | Clearly define all outcomes, exposures, predictors, potential confounders, and effect modifiers. Give diagnostic criteria, if applicable | 6–7 |
| Data sources/ measurement | 8 | For each variable of interest, give sources of data and details of methods of assessment (measurement). Describe comparability of assessment methods if there is more than one group | 5–6 |
| Bias | 9 | Describe any efforts to address potential sources of bias | 7–8 |
| Study size | 10 | Explain how the study size was arrived at | 5 |
| Quantitative variables | 11 | Explain how quantitative variables were handled in the analyses. If applicable, describe which groupings were chosen and why | 7–8 |
| Statistical methods | 12 | (*a*) Describe all statistical methods, including those used to control for confounding | 7–8 |
|  |  | (*b*) Describe any methods used to examine subgroups and interactions | 7–8 |
|  |  | (*c*) Explain how missing data were addressed | 7–8 |
|  |  | (*d*) If applicable, explain how loss to follow–up was addressed | 7–8 |
|  |  | (e) Describe any sensitivity analyses | 8 |
| Results | | |  |
| Participants | 13 | (a) Report numbers of individuals at each stage of study—eg numbers potentially eligible, examined for eligibility, confirmed eligible, included in the study, completing follow–up, and analysed | Figure S1 |
|  |  | (b) Give reasons for non–participation at each stage |  |
|  |  | (c) Consider use of a flow diagram |  |
| Descriptive data | 14 | (a) Give characteristics of study participants (eg demographic, clinical, social) and information on exposures and potential confounders | 9 |
|  |  | (b) Indicate number of participants with missing data for each variable of interest | Figure S1 |
|  |  | (c) Summarize follow–up time (eg, average and total amount) | 9 |
| Outcome data | 15 | Report numbers of outcome events or summary measures over time | 10, Table 2 |
| Main results | 16 | (*a*) Give unadjusted estimates and, if applicable, confounder–adjusted estimates and their precision (eg, 95% confidence interval). Make clear which confounders were adjusted for and why they were included | 10, Table 2 |
|  |  | (*b*) Report category boundaries when continuous variables were categorized | Table 2 |
|  |  | (*c*) If relevant, consider translating estimates of relative risk into absolute risk for a meaningful time period | Figure 2 |
| Other analyses | 17 | Report other analyses done—eg analyses of subgroups and interactions, and sensitivity analyses | 11, table 3 |
| Discussion | | |  |
| Key results | 18 | Summarise key results with reference to study objectives | 11 |
| Limitations | 19 | Discuss limitations of the study, taking into account sources of potential bias or imprecision. Discuss both direction and magnitude of any potential bias | 13–14 |
| Interpretation | 20 | Give a cautious overall interpretation of results considering objectives, limitations, multiplicity of analyses, results from similar studies, and other relevant evidence | 11–13 |
| Generalisability | 21 | Discuss the generalisability (external validity) of the study results | 12 |
| Other information | | |  |
| Funding | 22 | Give the source of funding and the role of the funders for the present study and, if applicable, for the original study on which the present article is based | 16 |
